# Dental Hygiene

**Published:** 1871-06

**Authors:** L. J. B. Leblanc


### ARTICLE IX.

### *Dental Hygiene.*

BY L. J. B. LEBLANC, L. D. S.

Translated from the French, and read before the Montreal Dental Society.

I have not the intention of writing a work of considerable length, but a mere essay on dental hygiene, and I shall abstain from those long technical words, more or less irregular; some would seem like India-rubber, on account of the facility with which they are lengthened at pleasure. My object is, therefore, to awaken the attention of the public on one of their most precious advantages, which in order to preserve them, they display the most incomprehensible indifierence. Nevertheless, this subject concerns, at the same time, the cleanliness, health and rest which are obtained only by the absence of pain and the most constant care. We do not agree, I believe, on what really constitutes beauty. Our different ways of seeing and judging render its definition an object of great difficulty. All the parts which compose the human fignre vary in their form to the utmost extent. That is why we are prevented from clearly defining it, and describing how their harmony produces what we agree to call a handsome or ugly face. So we are induced to say that beauty is only and properly regarded as such by a general and implied convention ; but that convention is unspecified according to different climates —for instance, a narrow forehead, thick lips, a broad flat nose, wool instead of hair, are the marks of beauty among Africans, and among us, signs of ugliness. But teeth are said to be handsome, when they are white and well arranged. When nature made them handsome, it took everywhere the same care of setting them in rosy guns ; and the carmine of the lips set off their whiteness still more. They are not only ornamental but also very useful to health. Many, however, seem to be ignorant of that important truth. Nevertheless the slightest attention is needed to convince one's self that teeth are absolutely necessary to the preservation of the animal economy, since they are designed for one of our principal functions. How many teeth were broken by imprudence and vain show, especially among those set for the trituration of food, and consequently for the easy digestion of the stomach, which is only the secondary agent and above all the protector of health ; hence if it is deprived of the preparatory work of the jaw, and receiving but food half triturated, its various functions are laborious—wasting slowly its elasticity, it grows weak, and loses that vigor which, when once lost, cannot be restored by the most powerful tonics, and the richest of victuals. "You are a foe to your life if you do not masticate well" says the latin proverb. Those who have good teeth and do not masticate well can profit by this lesson. But what can we say to those who have bad ones ? They must guard against that difficulty by dint of care, since a good mastication is so necessary to health. Yes, when the uneasiness, the bad digestion and the weakness of the stomach set in, one says: "If I had my teeth I would give a great deal;" but when you had them—it was perhaps troublesome, but it is far more troblesome at present. A physician said very wittily: "Formerly the fable of the stomach and its parts was composed, but if at present we wrote that of the stomach and the teeth—oh! how numerous would be the wrongs of the latter towards the former." The same writer attributes rightly a great number of diseases of which the cause is unknown, to the impurities that the saliva of slovenly mouths carry in the blood, bringing them along by mastication. This causes at length a bad chyle always injurious to health.

You can see, gentlemen, that the teeth are as necessary to health as to beauty; it is for that reason that we always seek the means of keeping them sound. The process for that aim is ordinarily slow. It is submitted to general rules which I will explain as briefly as possible.

Generally the teeth of the first dentition are not susceptible of any care for cleanliness, unless they be effected by decay, in which case they must be plugged, and brushed often in order to check the progress of that affection. It is at the age of seven or eight that children should be taught the habit of brushing their teeth twice or three times a week, with a soft brush, so as to provide against caries, and the pains, more or less smarting, resulting from it. By these means the teeth and the mouth are kept in a state of cleanliness and freshness which is so agreeable. In case the teeth should be covered with tartar, it would be necessary to remove it to avoid caries or inflammation of the gums. Another inconvenience of tartar is that it causes an offensive breath.

From the age of fifteen the teeth must be brushed every day, and with a powder well prepared, two or three times a week. The remainder of the times with a liquid acting like a tonic, which will dissolve in the same time the mucus deposited on our teeth while sleeping. Experience teaches us, gentlemen, that their daily cleaning is their best preservative. It would be more suitable to clean them after meals in order to remove the alimentary substances; and if particles of food are deeply placed in them, a tooth-pick should be used, a custom generally known at the present. There are parties who satisfy themselves with a piece of linen to rub their teeth without taking the precaution of rinsing the mouth. This is far from being the clean and proper way; on the contrary, it is very injurions, because a certain pressure is made on the gums, and that habit has no other advantage but that of gathering and hardening tartar in places where it is liable to accumulate itself, that is to say, between the teeth and their necks.

Now, persons wearing false teeth are not absolved from cleanliness, more than others; they should take great care of the mouth; if otherwise, the sets cover themselves with food and tartar, which remaining habitually in moist and warm places, become infected and cause a great deal of inflammation to the mucous parts, and then the mouth becomes the seat of an intolerable smell. Unfortunately, gentlemen, we have often the evidence of what I have just said. A man must despise himself to a great extent to keep so much dirtiness, and we, poor dentists, are often compelled to stand a good distance from these persons, and in spite of us, their breath, which is able to kill flies while flying, reaches us; we would not be so unlucky if nature had given them, as the alligators of San Domingo, under their lower jaw, the advantage of having a gland holding musk.

Though the rubbing of the teeth with a mere brush dipped in aromatized water is nearly always sufficient for maintaining the cleanliness of these organs, there are nevertheless persons either by the nature of their constitution or by previous negligence, who are compelled to seek more energetic means, that is to say making a great use of powders.

I shall say a few words on those which are still employed at present. Coal well ground, is a popular dentifrice; it is an antiseptic; its use almost given up, still they prepare it in our drug stores; its action is nearly useless on the enamel —its constant use ends by causing the teeth to appear black and the gums as afflicted with scurvy. This applies also to burnt crusts of bread which are used by several persons. Soot has been used for some time because it was believed that the chimney sweep's teeth were always white—that belief is erroneous. Their teeth appear white because their faces are black—as for instance, with negroes it is the mere contrast of the color of their skin. Notwithstanding, the latter possess good, handsome and strong teeth. Its use is utterly unclean. Cinchona is a torpid powder, but its taste and color on the one hand, and on the other its tanning principle, which at length make the enamel yellow, cannot recommend it as a dentrifice. It has, however, the property of hardening the gums. What I have said of cinchona can also be appied to tobacco.

*Salt* is by no means injurious, but it determines a considerable secretion of saliva.

Cigar ashes are also in use, that powder is very unwholesome—it is too strong an alkali. Alum, that substance which we can rank with tartaric and oxalic acid, are too strong powders, unless in order to use them we mix them with an absorbing substance having the property of neutrallizing their acidity. We must mistrust those patented liquids and powders, the pompous names of which fill the columns of our newspapers, ornament the corners of our streets, and are used as mile-posts on our country roads. Remember well, the action of these patented drugs is magic—it is exactly there where lies the evil, because the only dentifrices which can give quickly a shining white ivory-looking appearance to the teeth are acid. The calcareous phosphate, which is the base of the enamel, is dissolved, causing its polish to be removed, and the teeth thereafter are more liable to retain that kind of mucus which previously had a tendency to accumulate on them. They assume an indelible yellow tint, and if you continue to use those compounds, if the acids constituting their bases are too concentrated, they will soon uncover the gelatinous substance of the teeth, which will cause aching, caries, and their extraction will become unavoidable,

Yes, gentlemen, the first quality of a dentifrice is to clean the teeth thoroughly and without injury.

1st. "Whatever may be the mode of preparation of those tooth powders," says Dr. Maury, "we must exclude from their composition all substances liable to alter the enamel of the teeth, since those sorts of preparations are to be used only to maintain their whiteness by removing the tartar which gathers on them.

2d. "You must note their action on the gums." Are those conditions observed in the dentifrices offered to the public with the most pompous names? I entertain great doubts about that. It is a mere speculation now-a-days, and the voice of interest very often stiflles that of humanity.

This induces me to say a few words on an article which each of you, I am sure, possess—tooth brushes. Each hair of them may be considered as a tooth-pick, the daily use of the brush cleans the teeth and the gums, and saves them from many diseases. Brushes vary a great deal in their form, that is why you must choose them. For children they must be straight and very soft, for adults they must be so much the less hard as the gums are softer. For instance, if you select a hard brush you erase thus the enamel in some ways, you lacerate and bleed the gums, you uncover the non-enameled substances of the teeth, they will totter, sensibility will ensue, and afterwards the toothache.

An article everybody should possess is a tooth-pick. It must be used only when a few particles of food are driven between the teeth, which they annoy when they cannot be taken off in spite of the efforts of the tongue. That is the only circumstance when we can rationally recommend the use of a tooth pick. The teeth and gums should be tormented the least possible with that instrument or any similar object.

There is still another error which I must note. It is the use of the end of a penknife as a tooth-pick. By that process a few blades of the enamel are exposed to be cut off, and unfortunately those occurrences take place and caries, act in that place. Next, are you always sure that your knife is clean? This is again a very delicate matter, because while cleaning your teeth with that instrument you can wound the gums and bring on an inflammation. The best toothpicks are not made from gold, silver or steel, but those which are manufactured from quills.

Formerly sponge and cotton plugs were used for cleaning the teeth—this habit was far from being suitable for keeping the mouth clean—they had the same disadvantages as the linen of which I have already said a few words. Independently of the hygienic care required by the teeth, there are some other precautions to be taken in order to preserve the beauty and soundness of those organs, and those precautions consist in avoiding carefully all that is injurious or may become so. As there are many I shall select the most remarkable, and I will be as brief as possible.

1st. You must avoid washing your head in water too cold or too warm, using those astringent and caustic remedies to remove freckles, or dying the hair.

2d. Not to break with your teeth too hard objects, or cutting threads or any other thing with the *incisive teeth*, for they become notched and decayed.

3d. Never leave particles of food in these organs, nor use too hard dentifrice powders, or elixirs, tinctures too much acidulated.

4th. While eating, avoid taking food or beverages too warm or too cold, because the sudden transition between those two extremes is always injurious to the teeth. *Frigidus inimicum dentibus*, said Hippocrates with reason. When we smoke a pipe or cigar, the mouth is warmed, the larynx becomes dry, and then you drink—and even sometimes very cold water that may cause a slight inflammation of the dental pulp.

5th. Be very careful when drinking mineral waters, for they can irritate them, render them painful, make them turn yellow, and give them a dark covering.

Abstain from sweet things: a proof that they are injurious is that I have met in my practice, confectioners, still young, whose teeth were nearly all carious. Druggists come under the same heading, because frequently they are obliged to taste their preparations, acid or sweet.

A thing that is very often done and about which we are careless, is drinking in the same glass as another, or smoking from the same pipe—that is far from being clean. It occurs often that an impure saliva left on the edge of a glass, poisoned those who drank from it afterwards. Sailors are less particular when their officer calls them while they are enjoying a *chew*, they don't care about handing it to a comrade who masticates deliciously before returning it to its proprietor.

6th. Do not be great lovers of crackers and biscuits, they always contain acid powders. Americans have generally very bad teeth—we may say that they are the greatest biscuit eaters of the world.

7th. Abstain as much as possible from the use of gaseous waters, such as soda, which is relished so much in the hot season—it is acid, since it is composed from tartaric acid and bi-carbonate of soda. Ice-cream is also very injurious. As to myself I must confesss that I am a great lover of it; do what I tell you and not what I do, as a proverb says. Salads are unwholesome: notwithstanding, taken seldom and in small quantities, they can stimulate appetite and facilitate digestion.

8th. Feats of strength performed with the teeth are absurd; those who indulge in them ought to be punished like that youth, who, says Dr. Lemartie, broke all his front teeth who bet that he would throw over his head a chair which he held with his teeth by the upper part of the back board to achieve that noble feat. Another fellow, more imprudent, caused himself to be hoisted up from the ground to an window by means of a rope which he held in his teeth. When he reached a certain height he lost his four incisors and broke one of his legs in the fall. Some others, says the Doctor, find pleasure in grinding drinking glasses between their teeth and wounding their mouths grievously in the attempt—one would suppose that the life of these maniacs is a perpetual challenge to the Almighty who gave it to them. The loss of a tooth is a real misfortune since it cannot be repaired A tooth is worth a diamond says one of our authors. Remember these few word, gentlemen, and try to put them in practice, and when old age will come, your teeth will still be handsome. If you do the contrary your cheeks will sink in, your lips will lose their firmness and embrossment, your chin will be lengthened, wrinkles will cover your face as so many furrows, pronunciation will be difficult and unpleasant, the saliva, having no more dykes to contain it, will escape, and produce that unpleasantness which we hardly endure in old persons.

Before concluding, I must tender you my best thanks for your kind attention. I have endeavored as much as possible to be brief and varied, to avoid the aridity and monotony which are usually found in medical works, where one's health is treated in a grave and doctorial style; I am satisfied after saying only what could be useful. I will be doubly rewarded if I succeed in giving some credit to this essay, and in deserving a smile of approbation from those for whom I undertook especially to compose it.—*Canada Journal of Dental Science.*

---

